# Small One-Helix Proteins Are Essential for Photosynthesis in Arabidopsis

**DOI:** 10.3389/fpls.2017.00007

**Published:** 2017-01-23

**Authors:** Jochen Beck, Jens N. Lohscheider, Susanne Albert, Ulrica Andersson, Kurt W. Mendgen, Marc C. Rojas-Stütz, Iwona Adamska, Dietmar Funck

**Affiliations:** Plant Physiology and Biochemistry Group, Department of Biology, University of KonstanzKonstanz, Germany

**Keywords:** Early light-induced protein family, phylogeny, pigment-protein complexes, photosynthesis, photoprotection

## Abstract

The extended superfamily of chlorophyll *a/b* binding proteins comprises the Light-Harvesting Complex Proteins (LHCs), the Early Light-Induced Proteins (ELIPs) and the Photosystem II Subunit S (PSBS). The proteins of the ELIP family were proposed to function in photoprotection or assembly of thylakoid pigment-protein complexes and are further divided into subgroups with one to three transmembrane helices. Two small One-Helix Proteins (OHPs) are expressed constitutively in green plant tissues and their levels increase in response to light stress. In this study, we show that OHP1 and OHP2 are highly conserved in photosynthetic eukaryotes, but have probably evolved independently and have distinct functions in Arabidopsis. Mutations in *OHP1* or *OHP2* caused severe growth deficits, reduced pigmentation and disturbed thylakoid architecture. Surprisingly, the expression of OHP2 was severely reduced in *ohp1* T-DNA insertion mutants and *vice versa*. In both *ohp1* and *ohp2* mutants, the levels of numerous photosystem components were strongly reduced and photosynthetic electron transport was almost undetectable. Accordingly, *ohp1* and *ohp2* mutants were dependent on external organic carbon sources for growth and did not produce seeds. Interestingly, the induction of ELIP1 expression and Cu/Zn superoxide dismutase activity in low light conditions indicated that *ohp1* mutants constantly suffer from photo-oxidative stress. Based on these data, we propose that OHP1 and OHP2 play an essential role in the assembly or stabilization of photosynthetic pigment-protein complexes, especially photosystem reaction centers, in the thylakoid membrane.

## Introduction

Photosynthetic energy conversion in plants and algae forms the basis for almost all life on earth. Oxygenic photosynthesis most likely evolved in ancestral cyanobacteria around 2.7–3.5 billon years ago (Nowicka and Kruk, 2016). More than 1.5 billion years ago, engulfment of a cyanobacterium by a eukaryotic host cell gave rise to chloroplasts and thereby photo-autotrophic eukaryotes (Archibald, 2015). During the co-evolution of plastids and their host cells, the major part of the organelle genome was transferred to the host nucleus, generating the need for protein import to maintain organelle structure and function (Zimorski et al., 2014). Early on, the photosynthetic eukaryotes split into several lineages, giving rise to the now living Glaucophytes, Chloroplastida, and Rhodophytes. In both the “green” and the “red” lineage, further endocytobiosis events gave rise to secondary or even tertiary plastids (Zimorski et al., 2014).

A common feature of all photosynthetic organisms is that the conversion of light energy into chemical energy is performed by large pigment-protein complexes in specialized membrane systems, the thylakoids (Nelson and Ben-Shem, 2004). In plants, the thylakoids are structurally and functionally heterogeneous: Grana stacks predominantly contain the water-oxidizing photosystem II (PSII) while stroma lamellae harbor PS I and the ATP-synthase complex (Pribil et al., 2014). Assembling the photosynthetic complexes in the thylakoid membrane requires a tight coordination of protein synthesis and folding with pigment synthesis and delivery. Failures of this coordination can lead to protein mis-folding or accumulation of uncoupled pigments that initiate deleterious processes rather than funneling the absorbed energy to the photosynthetic reaction centers (Rochaix, 2011; Komenda et al., 2012; Wang and Grimm, 2015).

Recent studies on cyanobacteria demonstrated that the family of High Light Induced Proteins (HLIPS) plays important functions as carriers of newly synthesized pigments during the assembly of photosystem II and potentially also photosystem I (Chidgey et al., 2014; Knoppová et al., 2014; Akulinkina et al., 2015). As their name implies, HLIPs were identified by their up-regulation during high light stress, when photo-damaged photosystems have to be degraded and replaced. In this process, HLIPs may additionally function in the recycling of pigments from damaged photosystems or antennae (Dolganov et al., 1995; Havaux et al., 2003).

HLIP-like proteins are also present in all photosynthetic eukaryotes and are probably the evolutionary ancestors of the Light Harvesting Complex (LHC) superfamily of proteins (Engelken et al., 2012). The common feature of all LHCs and LHC-like proteins are conserved transmembrane helices that mediate the binding of chlorophyll (Chl) and carotenoids. In higher plants, the LHC superfamily contains members with one to four transmembrane helices. Parallel to the structural diversification, also the function of the proteins has changed. The three-helix LHCs form the membrane-integral inner and outer antennae of both photosystems and thus contribute to the efficiency of light harvesting (Büchel, 2015). In contrast, the four-helix protein PSBS is an important factor in the induction of excess energy dissipation within the LHC antenna (Li et al., 2000; Niyogi and Truong, 2013). Another class of three-helix proteins, the Early Light Induced Proteins (ELIPs) are almost exclusively expressed during light stress and de-etiolation of proplastids. They do not contribute to light harvesting but may act as pigment carriers during the assembly of photosynthetic complexes (Montané and Kloppstech, 2000; Adamska, 2001). Mutant analyses revealed only a minor function of ELIPs in light stress tolerance, whereas they contributed to the regulation of germination (Casazza et al., 2005; Rossini et al., 2006; Rojas-Stütz, 2008; Rizza et al., 2011). The two-helix LHC-like (LIL) or Stress Enhanced Proteins (SEPs) were proposed to function in photoprotection (Heddad et al., 2012). For two recently diverged LIL3 isoforms in Arabidopsis, an important function in stabilizing or localizing protein complexes mediating late steps in Chl biosynthesis has been shown (Tanaka et al., 2010; Takahashi et al., 2014; Lohscheider et al., 2015).

The One-helix proteins (OHPs) resemble most closely the ancestral cyanobacterial HLIPs and almost all photosynthetic eukaryotes contain at least one member of each of the two sub-classes OHP1 and OHP2 (Engelken et al., 2012). Based on the gene expression patterns of Arabidopsis *OHP1* (At5g02120) and *OHP2* (At1g34000) and the localization of both OHP1 and OHP2 in thylakoid membranes, functions in light stress protection and de-etiolation of proplastids have been proposed (Jansson et al., 2000; Andersson et al., 2003; Stawski et al., 2014). In this study, we analyzed the evolutionary relationship between OHP1 and OHP2 and directly compared their gene expression patterns under various conditions. To unravel their functions *in planta*, we characterized *ohp1* and *ohp2* T-DNA insertion mutants. Surprisingly, we found that *ohp1* and *ohp2* mutants of Arabidopsis showed nearly identical phenotypes with chlorotic appearance, high Chl fluorescence, strongly decreased levels of photosystems resulting in the prevention of photoautotrophic growth and altered thylakoid ultrastructure. Complementation analyses confirmed the specificity of the mutant phenotypes and demonstrated that OHP1 and OHP2 have essential, non-redundant functions in Arabidopsis.

## Materials and methods

### Phylogenetic analysis

Sequence data were collected from NCBI databases (http://www.ncbi.nlm.nih.gov), protein sequences were aligned using the ClustalW algorithm (Larkin et al., 2007) and the alignment was manually refined in Bioedit (Hall, 1999). Phylogenetic analysis was performed with PHYML (http://www.atgc-montpellier.fr/phyml) with 100 bootstrap replicates (Guindon et al., 2010).

### Plant material and growth conditions

*Arabidopsis thaliana* (L.) Heynh., ecotype Col-0 was obtained from the NASC (Stock-Nr. N60000) and T-DNA insertion mutants *ohp1-1* (GABI_362D02, N434694), *ohp1-2* (GABI_631G03, N460555), and *ohp2-1* (GABI_071E10, N406778) were obtained from the GABI-KAT project (Kleinboelting et al., 2012). Surface-sterilized seeds were grown on Murashige and Skoog plant medium (Duchefa Biochemie, Harleem, The Netherlands) solidified with 0.8% (w/v) agar and supplemented with 3% (w/v) sucrose (unless stated otherwise), with or without 10 mg L^−1^ sulfadiazine. Plants were grown in a growth chamber under either continuous illumination or a 8 h light/16 h dark cycle with light intensities of 10 to 100 μmol photons m^−2^ s^−1^ at 23°C (±2°C). Homozygous and heterozygous mutants were identified by PCR on genomic DNA using gene-specific primers in combination with a T-DNA specific primer (Supplemental Table 1). For transcript and protein analyses, wildtype (WT) plants were grown in individual pots in a phytochamber with an 8 h day/16 h night cycle at 21/17°C and a light intensity of 120 μmol photons m^−2^ s^−1^ at a relative humidity of 60%. Heterozygous mutants for seed production were grown on soil in a greenhouse with at least 16 h of light per day.

### Isolation of genomic DNA and northern blotting

Genomic DNA was extracted from leaves using the High Pure GMO Sample Preparation Kit (Roche, Basel, Switzerland) according to the manufacturer's instructions. Total RNA was isolated from frozen leaf material (−80°C) using Trizol (Thermo Fisher Scientific, Carlsbad, CA, USA) and the RNeasy Kit (Qiagen, Hilden, Germany). Northern blots were carried out using the DIG labeling and detection system (Roche, Basel, Switzerland). Labeled probes comprising the entire CDS of *OHP1* or *OHP2* or approximately 300 bp of *ELIP1* or *ACT2* were generated by PCR, column-purified and diluted in high-SDS hybridization buffer (see Supplemental Table 1 for primer sequences). RNA separation, transfer to a nylon membrane (Pall Corp., Port Washington, NY, USA), hybridization and detection were performed as described by Woitsch and Römer (2003).

### Mutant complementation

The CDSs of *OHP1* or *OHP2* were inserted by InFusion cloning (Takara Bio Europe, Saint-Germain-en-Laye, France) into a pEG100-derived plant transformation vector containing a synthetic riboswitch in the 3′-UTR of the 35S-driven expression cassette (Earley et al., 2006; Ausländer et al., 2010). *Agrobacterium tumefaciens* strain GV3101 was used to introduce the constructs into heterozygous *ohp1-1* or *ohp2-1* mutants by floral dip (Clough and Bent, 1998).

### Protein isolation and analysis

Plant material was frozen in liquid nitrogen and crushed using either mortar and pestle or a Tissue Lyzer (Qiagen, Hilden, Germany). Samples were suspended in 50 mM Tris-HCl pH 8, 5 mM MgCl_2_ and centrifuged at 16,000 *g* for 10 min at 4°C. Soluble and membrane-associated proteins were removed by washing the pellet with 50 mM Tris-HCl, pH 7.5 supplemented with increasing NaCl concentrations of 0, 250, and 500 mM. Chl was removed by washing the pellet with 80% (v/v) acetone. For denaturing gel electrophoresis, membrane proteins were solubilized in sample buffer containing 50 mM Tris-HCl, pH 7.5, 2% (w/v) LDS, 50 mM DTT, 0.01% (w/v) bromphenol blue and 10% (v/v) glycerol for 30 min at 45°C (modified from Leto and Young, 1984). Protein concentrations were determined using the RC/DC Protein Determination Kit (Bio-Rad, Hercules, CA, USA). For efficient separation of proteins bigger than 15 kDa, SDS-PAGE was performed according to Laemmli (1970) using Biorad Minigel Systems (Bio-Rad, Hercules, CA, USA). For the analysis of smaller proteins, Tris-Tricine buffered SDS-PAGE was performed as described by Schägger and von Jagow (1987). Ten or twenty microgram protein was loaded per lane.

Immunoblotting was carried out according to Towbin et al. (1979) using polyvinylidene difluoride (PVDF) membranes with 0.45 μm pore size (GE-Healthcare, Little Chalfont, UK) at a current of 0.5 mA cm^−2^ of membrane for 40 min for small proteins (below 15 kDa) and 1 mA cm^−2^ of membrane for 60 min for proteins above 15 kDa. Membranes were blocked in 5% (w/v) non-fat dry milk dissolved in PBS containing 0.1% (v/v) Tween 20. Horseradish peroxidase-coupled secondary antibodies and enhanced chemiluminescence reagents (ECL Plus, GE Healthcare, Little Chalfont, UK) were used as a detection system. Antibody sources: anti-OHP1 (see below), anti-OHP2 (Andersson et al., 2003), anti-ELIP1 (Heddad et al., 2006), anti-33 kDa protein of the oxygen-evolving complex (PSBO; Lundin et al., 2008), all other antibodies were obtained from Agrisera AB (Vännäs, Sweden).

Green-Native PAGE of total protein extracts from entire rosettes was performed according to Allen and Staehelin (1991).

### Recombinant expression of OHP1 and production of polyclonal antibodies

The *OHP1* coding sequence was amplified with specific primers (Supplemental Table 1) from cDNA and inserted into the *E. coli* expression vector pBAD/Thio-TOPO (Thermo Fisher Scientific, Carlsbad, CA, USA). Overexpression was performed according to the manufacturer's protocol. The fusion protein accumulated in inclusion bodies that were purified according to Chen et al. (1991), resuspended in 10 mM NaHPO_4_ pH 7.2, 0.9% (w/v) NaCl and used to raise a polyclonal OHP1-antiserum in rabbit (TFA, University of Konstanz, Konstanz, Germany). The antiserum reacted strongly with recombinant OHP1 but showed cross-reactivity with another thylakoid protein that migrated to the same position in SDS gels.

### Pigment isolation and analysis

Plant material was processed as for membrane protein extraction, except that pigments were extracted by vortexing and sonication in the presence of 80% acetone. Cell debris was removed by centrifugation for 10 min at 16,000 *g* and 4°C. The absorbance of extracts was determined at 662 nm, 645 nm and 470 nm and the pigment content was calculated as described by Lichtenthaler (1987). To analyze pigment composition, extracts normalized on fresh weight were analyzed by thin layer chromatography. The stationary phase consisted of TLC aluminum sheets coated with silica gel 60. Pigments were separated in a mobile phase consisting of petroleum ether:diethyl ether:chloroform:methanol:acetone at the ratio 8:2:2:1:1.

### Electron microscopy

Rosette leaves of 6-week-old Arabidopsis plants were cut into small pieces of 2 × 3 mm and pre-fixed immediately with 2% (v/v) glutaraldehyde in 0.1 M phosphate buffer, pH 7.2, for 2 h at room temperature. Samples were then rinsed five times and post-fixed with 1% (w/v) osmium tetroxide for 2 h in the same buffer at room temperature. After dehydration in a graded ethanol series, samples were infiltrated and embedded in a mixture of Spurr's and Epon/Araldite resins. After polymerization, ultrathin sections of about 50 nm thickness were cut with a diamond knife and mounted on copper grids. Sections were stained with uranyl acetate first and post-stained with aqueous lead citrate (0.1 M, pH 13). Micrographs were taken with a Hitachi H 7000 electron microscope at 75 kV (Hitachi, Tokyo, Japan).

### Pulse amplitude modulated (PAM) fluorimetry

Chl fluorescence was monitored using an Imaging PAM Fluorometer (Walz GmbH, Effeltrich, Germany) equipped with a standard measuring head using the ImagingWin software provided by the supplier. Program settings used in kinetics experiments were: measuring light intensity 1, measuring light frequency 1, actinic light intensity 1 or 11 (corresponding to 8 or 150 μmol photons m^−2^ s^−1^, respectively), actinic light width 0, damping 2, gain 5, saturating pulse intensity 10, yield filter 3 and Fm-factor 1.024. Before the measurements, single leaves or whole plants were dark-adapted for 5 min and exposed to a light regime consisting of one saturating flash in the dark-adapted state followed by a period of actinic illumination with further saturating flashes until the photochemical quantum efficiency (ΦPSII) reached a steady state. After each measurement, absorptivity of the leaves or seedlings was estimated as suggested by the ImagingWin software and photosynthetic parameters were calculated by the software.

### Detection of reactive oxygen species and SOD activity

H_2_O_2_ and ${\text{O}}_{2}^{-}$ were detected *in situ* by infiltration of excised mature leaves with 3,3-diaminobenzidine or NBT solution, respectively, according to Thordal-Christensen et al. (1997) and Bournonville and Díaz-Ricci (2011) except that 0.02% (v/v) Silwet were included in the staining solutions. Superoxide dismutase (SOD) and catalase activities were estimated by in-gel activity staining according to Beauchamp and Fridovich (1971) and Clare et al. (1984). Entire rosettes were ground in 10 ml/g ice-cold extraction buffer [100 mM KHPO_4_ pH 7.5, 1 mM DTT, 3 mM EDTA, 0.4% (v/v) Triton X-100]. Soluble proteins were obtained after centrifugation at 25,000 g for 10 min at 4°C and quantified with Bradford's reagent. Per lane, 30 μg (for SODs) or 10 μg (for catalase) of total protein was separated on native polyacrylamide gels. After staining, digital images of the gels were processed with the ImageJ software (Schneider et al., 2012).

## Results

### Two distinct types of OHPs are present in plants

**Figure 1:**
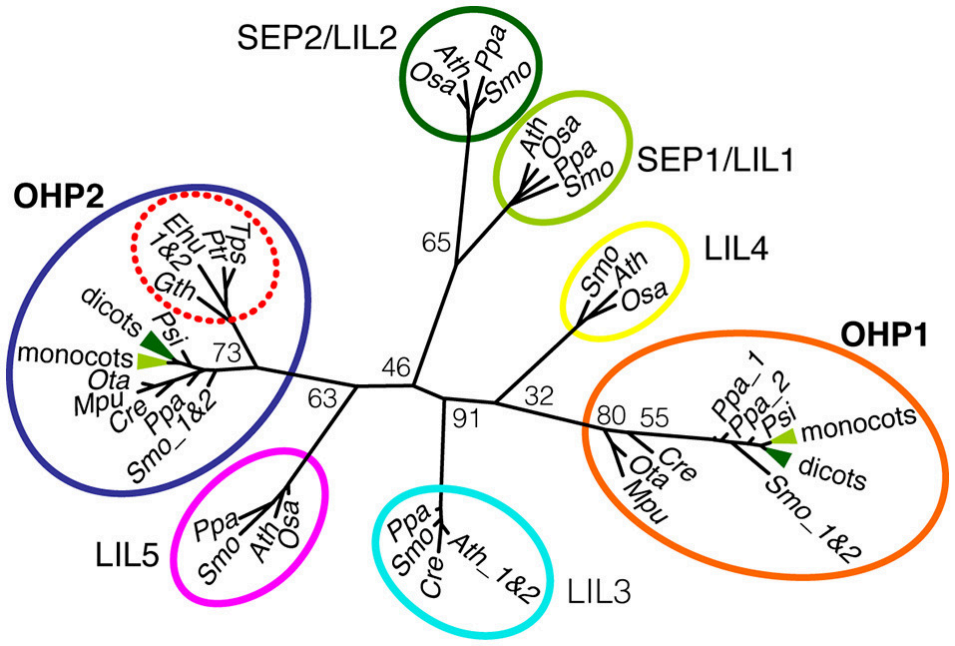
**Phylogenetic analysis of OHP1 and OHP2**. OHP and SEP/LIL sequences from selected plants and algae were aligned and used to calculate an unrooted maximum likelihood tree (See Supplemental Figure 1 for an excerpt of the alignment). Numbers at branching points are bootstrap values from 100 replicates. OHP1 and OHP2 proteins belong to two evolutionary distinct groups that most likely evolved independently in photosynthetic eukaryotes. Notably, secondary algae in the red lineage only contain OHP2 sequences (red dotted circle) but no nuclear-encoded OHP1. For a better overview, the highly conserved dicot (dark green) and monocot (light green) sequences were collapsed in the OHP1 and OHP2 branches of the tree and SEP/LIL sequences were only included from a small group of green algae and land plants. In addition to the species mentioned, OHP1 and OHP2 sequences from the dicots apple (*Malus domestica*), barrel clover (*Medicago truncatula*) and poplar (*Populus trichocarpa*) were included for the calculation of the tree. Seed plants: *Ath, Arabidopsis thaliana*; *Pop, Populus trichocarpa*; *Zma, Zea mays, Osa, Oryza sativa*; *Psi, Picea sitchensis*. Lower land plants: *Ppa, Physcomitrella patens*; *Smo, Selaginella moellendorffii*. Green algae: *Cre, Chlamydonomas reinhardtii*; *Ota, Ostreococcus tauri*; *Mpu, Micromonas pusilla*. Diatoms: *Ptr, Phaeodactylum tricornutum*; *Tps, Thalassiosira pseudonana*. Haptophyte: *Ehu, Emiliana huxleyi*. Cryptophyte: *Gth, Guillardia theta*.

Phylogenetic analyses based on the signature element of ELIP sequences, the conserved transmembrane helices with a predicted chlorophyll (Chl) binding motive, had very little power to resolve the evolutionary origin of the sub-families of OHP and SEP/LIL proteins (Engelken et al., 2012). We used a manually refined alignment of OHP and SEP/LIL sequences around the conserved transmembrane helix corresponding to the entire length of mature OHP1 to calculate a maximum likelihood tree (Figure 1 and Supplemental Figure 1). In this tree, OHP1 and OHP2 sequences are clearly assigned to separate branches, while the bootstrap values for the individual branches between the OHP and SEP/LIL proteins are too low to deduce an exact genealogy. Within each branch, the topology roughly follows the species tree of *Viridiplantae* (Rodríguez-Ezpeleta et al., 2005) with a separate branch of OHP2 sequences in organisms of the red algae lineage. In the red lineage, no nuclear-encoded OHP1 was identified, but they contain a putatively ancestral plastid-encoded High Light Induced Protein (HLIP) that is not present in the green lineage (Engelken et al., 2010). Among higher plants, OHP1 sequences and likewise OHP2 sequences are highly conserved and typically have more than 75% identical amino acids in the analyzed region within the monocot and dicot clades, whereas OHP1 sequences share only 10–20% identical amino acids with OHP2 (Supplemental Figure 1).

### Arabidopsis *OHP* genes are expressed constitutively and are upregulated upon light stress

**Figure 2:**
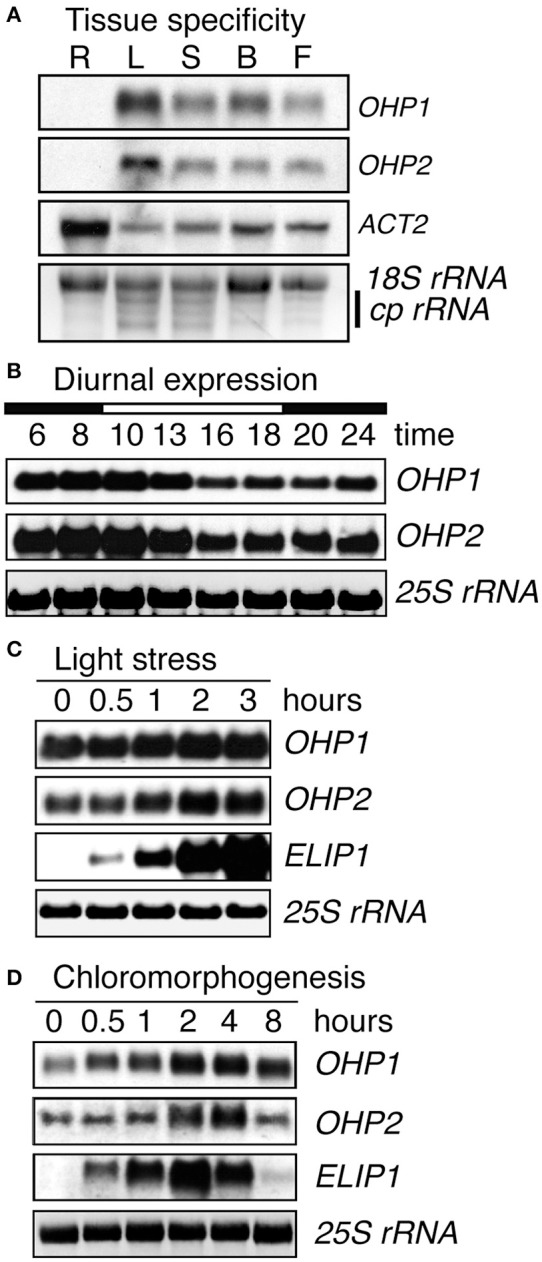
**Light regulation of *OHP1* and *OHP2* expression**. Transcript levels of *OHP1, OHP2, ELIP1*, and *ACT2a* in Col-0 (WT) plants were analyzed by northern blotting. **(A)** Different organs from mature plants in the reproductive phase were analyzed separately. R, Roots; L, Leaves; S, Stems; B, Buds; F, Flowers. Detection of *ACT2* demonstrates the successful isolation of mRNA from roots. EtBR-stained *18S rRNA* and chloroplast *rRNAs* (*cp rRNA*) are shown as loading control. **(B)** Diurnal regulation of *OHP1* and *OHP2* transcript levels in mature leaves of 6-week-old WT plants cultivated in an 8 h light/16 h dark cycle at a light intensity of 120 μmol photons m^−2^ s^−1^. The light period (10 a.m. to 6 p.m.) is indicated as a white bar on top of the time scale. **(C)** Light stress was induced by exposing detached leaves of plants cultivated at 120 μmol photons m^−2^ s^−1^ to a light intensity of 1000 μmol photons m^−2^ s^−1^. Induction of *ELIP1* expression served as a control to demonstrate the presence of light stress. **(D)** De-etiolation was induced by exposing 2-week-old, dark-grown WT seedlings to white light at an intensity of 100 μmol photons m^−2^ s^−1^. Again, expression of *ELIP1* served as a control to monitor the progression of de-etiolation. In **(B–D)**, EtBR-stained *25S rRNA* serves as loading control.

As expected for thylakoid membrane proteins, transcripts of both *OHP* genes showed the highest abundance in leaves and were present in all green plant tissues, whereas both *OHP1* and *OHP2* transcripts were undetectable in roots (Figure 2A). Diurnal regulation of *OHP1* expression had been described previously (Jansson et al., 2000). We observed that transcript levels of *OHP1* and *OHP2* showed very similar fluctuations during an 8 h light/16 h night cycle with the highest transcript levels before the onset and during the first half of the photoperiod being 25% higher than during the second half of the light phase and the beginning of the night (Figure 2B). Like other members of the ELIP family, OHP1 and OHP2 have been implicated in light stress responses (Jansson et al., 2000; Andersson et al., 2003). We confirmed that mRNA levels of both *OHP1* and *OHP2* were increased by approximately 40 and 60%, respectively, after 3 h of exposure to excess light (Figure 2C). In comparison to *ELIP1*, the increase in transcript levels was less pronounced for *OHP1* and *OHP2*, especially since both *OHP* genes showed much higher basal expression levels. *ELIP1* transcript levels were shown to increase during de-etiolation similarly to the response to light stress (Casazza et al., 2005). Also transcript levels of both *OHP1* and *OHP2* increased transiently by more than 100% during de-etiolation of dark-grown seedlings, peaking together with *ELIP1* after 2–4 h of exposure to light (Figure 2D).

Co-expression analysis of *OHP1* and *OHP2* using AttedII revealed that *OHP1* and *OHP2* expression is similarly regulated: both fall within the top 60 of co-expressed genes for each other (Obayashi et al., 2014). The top 100 lists of co-expressed genes for *OHP1* and *OHP2* share 22 common entries. Analysis of the complete ranked list of co-expressed genes for enriched GO terms for processes with GOrilla suggested a strong connection of *OHP1* and *OHP2* with glyceraldehyde-3-phosphate metabolism, isopentenyl pyrophosphate (IPP) metabolism and thylakoid membrane organization, along with other important chloroplast-associated processes (Eden et al., 2009).

### T-DNA insertion mutants of *OHP1* or *OHP2* show pale green phenotypes and are infertile

**Figure 3:**
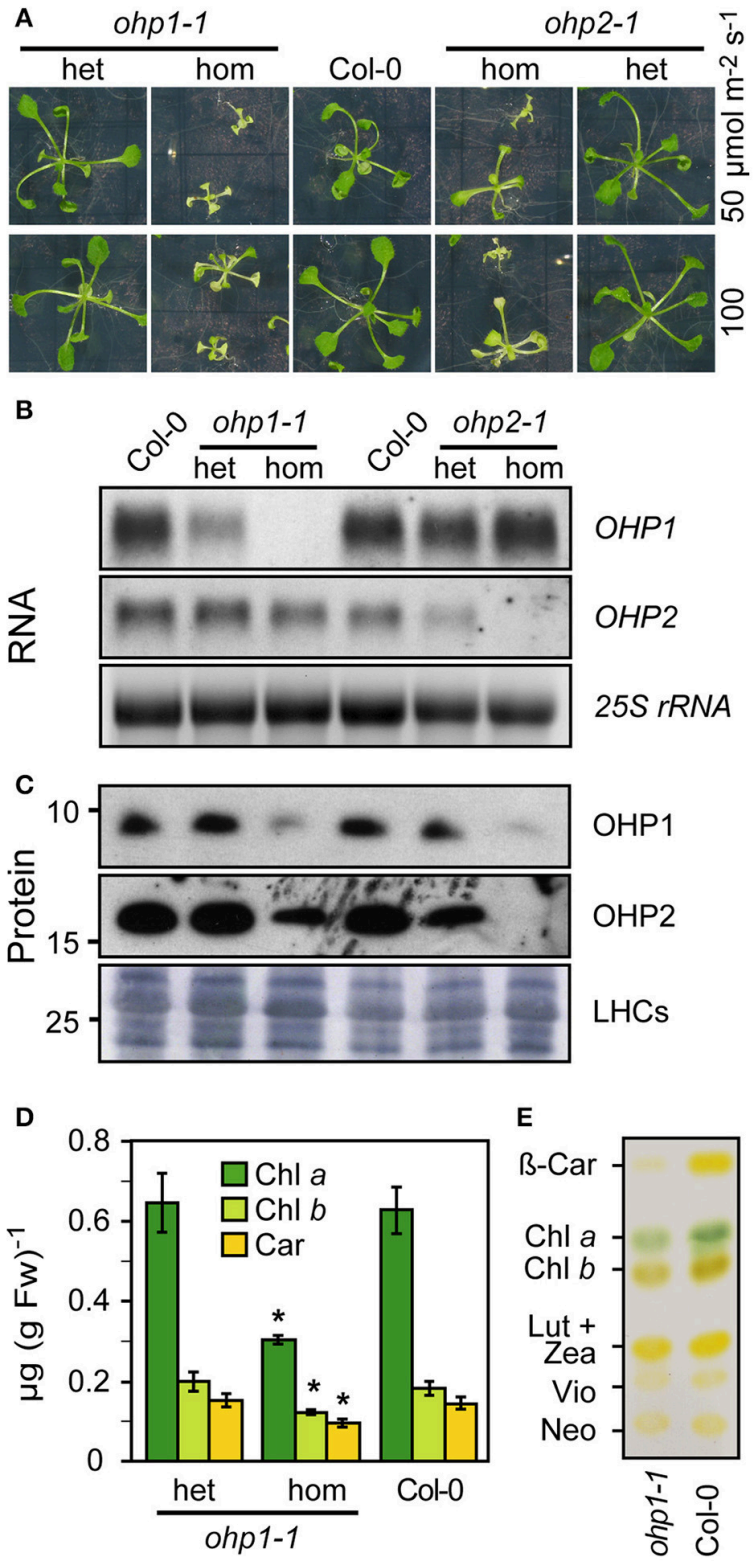
**Characterization of *ohp1* and *ohp2* mutants. (A)** Representative images of 3-week-old homozygous (hom) or heterozygous (het) *ohp1-1* and *ohp2-1* mutants in comparison to Col-0 WT plants of the same age. The plants were grown in axenic culture with continuous light. **(B)** Northern blot analysis of *OHP1* and *OHP2* transcript levels in the respective homozygous or heterozygous mutants in comparison to WT Col-0 plants. Fifteen microgram of total RNA were loaded per lane and EtBR-stained *25S rRNA* is shown as loading control. **(C)** Western blot analysis of OHP1 and OHP2 protein levels in corresponding samples. Ten microgram of LDS-solubilized insoluble proteins were loaded per lane and Coomassie-stained LHC proteins are shown as loading control. **(D)** Pigments from leaves of Col-0 WT plants or heterozygous and homozygous *ohp1-1* mutants were extracted with 80% acetone and quantified photometrically. Values represent the mean±SD (*N* = 4), Asterisks indicate significant differences from the respective WT value (*p* ≤ 0.01 by Student's *t*-test). **(E)** Pigments of homozygous *ohp1-1* mutants and WT seedlings separated by thin layer chromatography.

To unravel the function of OHP1 and OHP2 in thylakoids, we identified and characterized Arabidopsis T-DNA insertion mutants (Figure 3 and Supplemental Figure 2). The segregation of sulfadiazine resistance in the progeny of heterozygous plants of the T-DNA insertion line GABI_362D02 (*ohp1-1*) was consistent with a single T-DNA insertion (data not shown). As reported in the Chloroplast Function Database (Myouga et al., 2013), roughly one fourth of the progeny from plants heterozygous for the T-DNA insertion in *OHP1* stayed very small and were of pale green color (Figure 3A). PCR analysis of the pale green plants confirmed that they were homozygous for the T-DNA in the *OHP1* gene (Supplemental Figure 2). No *OHP1* transcripts could be detected in these plants (Figure 3B), demonstrating that *ohp1-1* is a complete loss-of-function mutant. A polyclonal antiserum raised against recombinant OHP1 detected residual amounts of a protein with the same molecular weight as OHP1 in protein extracts of homozygous *ohp1-1* mutants, indicating that the serum is not monospecific (Figure 3C). So far, we did not identify the cross-reacting protein. In MS-based protein detection of gel regions corresponding to the expected position of OHP1, three tryptic peptides of OHP1 were detected a total of 18 times (corresponding to 3.5% of all peptides identified unambiguously) in a wildtype (WT) sample (Supplemental Figure 2). In a corresponding gel slice with proteins from *ohp1-1* mutants, no OHP1-specific peptides were detected while peptides derived from other thylakoid proteins were detected with similar counts as in the WT sample, strongly suggesting the actual lack of the OHP1 protein in the *ohp1-1* mutant. Heterozygous *ohp1-1* mutants were indistinguishable from WT plants in size and coloration but they displayed reduced levels of *OHP1* mRNA (Figures 3A,B). In a second T-DNA insertion line, GABI_631G03 (*ohp1-2*), the T-DNA is located 72 bp upstream of the start codon of OHP1. Homozygous *ohp1-2* mutants showed low levels of residual *OHP1* expression and a similar but less severe mutant phenotype compared to *ohp1-1* mutants (Supplemental Figure 2D).

The line GABI_071E10 (*ohp2-1*) initially carried at least two T-DNA insertions. After backcrossing to Col-0 plants, a line with a single insertion in *OHP2* was identified. After self-fertilization, this line showed approximately 3:1 segregation of the sulfadiazine resistance marker and, surprisingly, homozygous *ohp2-1* mutants phenocopied *ohp1-1* mutants (Figure 3A). *OHP2* transcripts and protein were undetectable in homozygous *ohp2-1* seedlings (Figures 3B,C). The similar mutant phenotypes of *ohp1-1* and *ohp2-1* mutants indicated that OHP1 and OHP2 might act together in the same process despite their different evolutionary origin. Strikingly, hardly any OHP2 protein was detected in *ohp1-1* mutants and similarly, OHP1 protein levels were strongly reduced in *ohp2-1* mutants. In contrast, the transcript level of *OHP1* was not affected in *ohp2-1* mutants and likewise, *ohp1* mutants had unchanged transcript levels of *OHP2*.

The reduced growth and the pale color of *ohp1* and *ohp2* mutants indicated severe deficits in photosynthesis, which might cause problems during photosynthetically active phases of embryo development. Indeed, the occurrence of homozygous *ohp1-1* mutants was 34% less frequent than expected (Supplemental Figure 2G). Also among the progeny of heterozygous *ohp2-1* mutant plants, 28% less homozygous individuals than expected were observed, explaining the slightly lower than expected proportion of sulfadiazine-resistant plants. The defects of *ohp1-1* and *ohp2-1* seedlings in post-embryonic development could be a symptom of starvation as a result of impaired photosynthesis. In the absence of external sucrose, seedlings of both mutant lines were not able to develop beyond cotyledon stage, while increasing the sucrose concentration from 2 to 3% (w/v) improved the growth of the mutants (data not shown). Even in the presence of high sucrose concentrations, a developmental arrest and rapid bleaching of most *ohp1-1* and *ohp2-1* seedlings was observed in a day/night cycle with a light intensity of 100 μmol photons m^−2^ s^−1^. Illumination with very low light (10 to 15 μmol photons m^−2^ s^−1^), lowering the temperature from 23° to 19°C and/or supplementing the growth medium with Gamborg's vitamin mix allowed homozygous *ohp1-1* and *ohp2-1* mutants to develop further (data not shown). However, even under improved cultivation conditions photosynthetic performance remained very low. Not all homozygous mutants survived until flowering and none of them produced seeds (data not shown).

The modified growth regime allowed the generation of sufficient biomass of *ohp1-1* seedlings to investigate the pigmentation defect in more detail. Chl and carotenoids were extracted and quantified photometrically (Figure 3D). Confirming the visual impression, the pigment concentrations were very similar in WT and heterozygous *ohp1-1* mutants. In contrast, homozygous *ohp1-1* mutants had less than 50% of the Chl *a* concentration of WT seedlings and also Chl *b* and carotenoids were lower in *ohp1-1* mutants, although to a lesser extent than Chl *a* (Chl *b*: 68%, carotenoids 65%). The differential reduction in pigment concentrations was also reflected in altered pigment ratios. The Chl *a* to Chl *b* ratio and the total Chl to carotenoids ratio were reduced by 30 or 20%, respectively, in *ohp1-1* mutants as compared to WT plants. Thin-layer chromatography of isolated pigments demonstrated that mainly a decrease in β-carotene was the cause for the reduced carotenoid concentration in *ohp1-1* mutants, while xanthophyll levels were very similar compared to WT plants (Figure 3E).

### Chloroplast ultrastructure is altered in *ohp1* mutants

**Figure 4:**
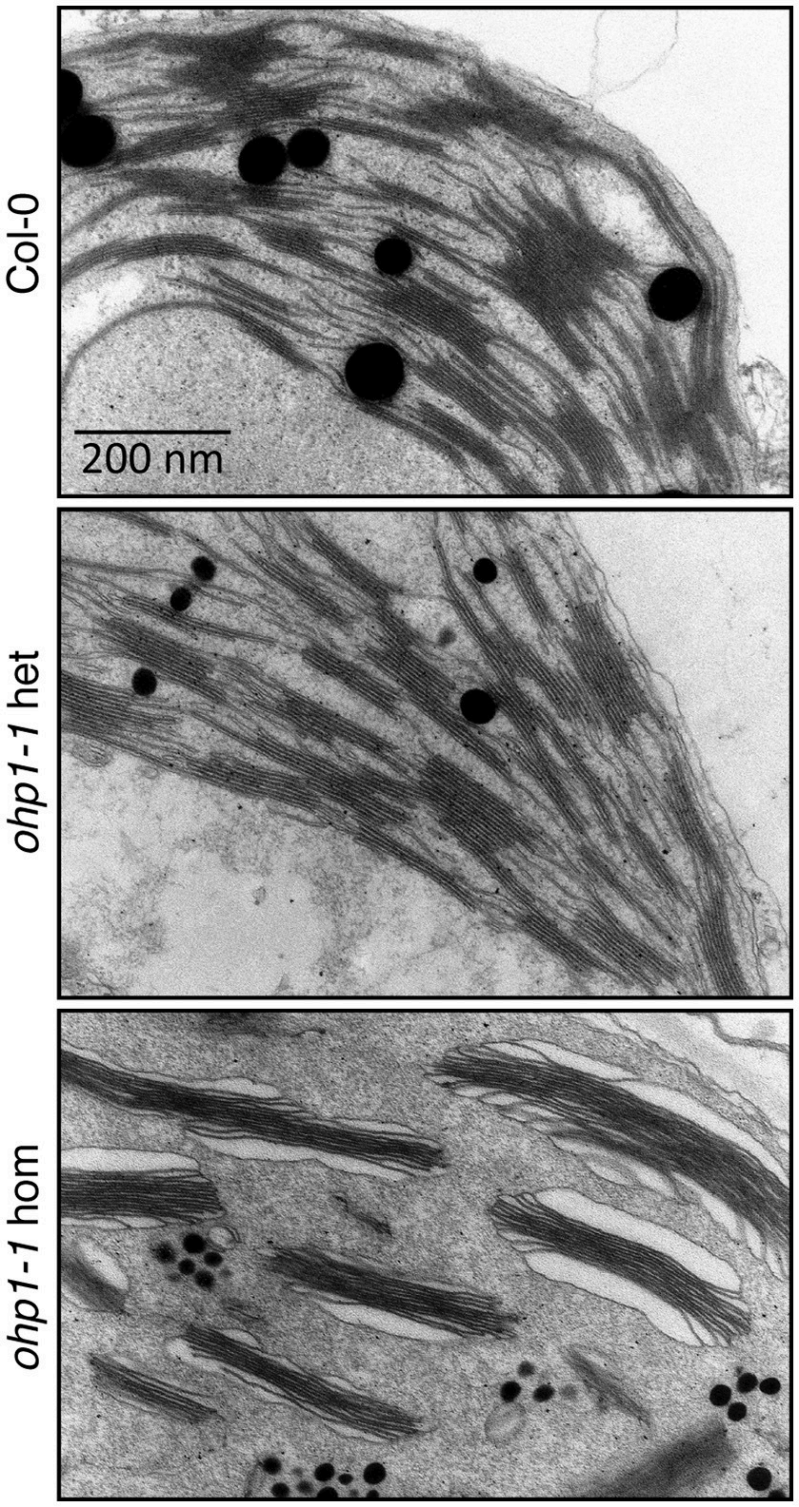
**Chloroplast ultrastructure is altered in *ohp1-1* mutants**. Mature leaves of 6-week-old plants cultivated at 15 μmol photons m^−2^ s^−1^ in an 8 h light/16 h dark cycle were analyzed by transmission electron microscopy. The micrographs show representative sectors of chloroplasts from WT plants in comparison to homozygous and heterozygous *ohp1-1* mutants. While heterozygous mutants have plastids with a similar appearance as the WT, homozygous *ohp1-1* mutants display strongly disturbed thylakoid ultrastructure.

In a previous study the pale green appearance of homozygous *ohp1* mutants in the L*er* background had been associated with altered chloroplast ultrastructure (Chloroplast Function Database, Myouga et al., 2013). To verify the requirement of OHP1 expression for normal thylakoid architecture, we analyzed the Col-0-derived mutant line *ohp1-1* by transmission electron microscopy (Figure 4). Micrographs of sections of true leaves showed identical pictures for WT plants and heterozygous *ohp1-1* mutants with well-differentiated grana stacks and stroma lamellae with interspersed plastoglobules. In contrast, stroma lamellae were virtually absent in leaves of homozygous *ohp1-1* mutants and plastoglobules appeared fragmented. Grana stacks were present in chloroplasts of *ohp1-1* mutants, but in non-appressed regions at grana stack margins the thylakoid lumen was strongly inflated. In comparison to the stroma, the content of the marginal thylakoids had a very low electron density, indicating that it contained only a low concentration of macromolecules. Additionally, starch grains were virtually absent in leaves of homozygous *ohp1-1* mutants, while they occurred with similar frequencies in plastids of WT plants and heterozygous *ohp1-1* mutants (data not shown). The structural aberrations observed in our specimens were different from the ones in the pictures contained in the Chloroplast Function Database, indicating ecotype- or tissue-specific effects of OHP1 depletion (Myouga et al., 2013).

### *ohp1* and *ohp2* mutants are impaired in photosynthesis

**Figure 5:**
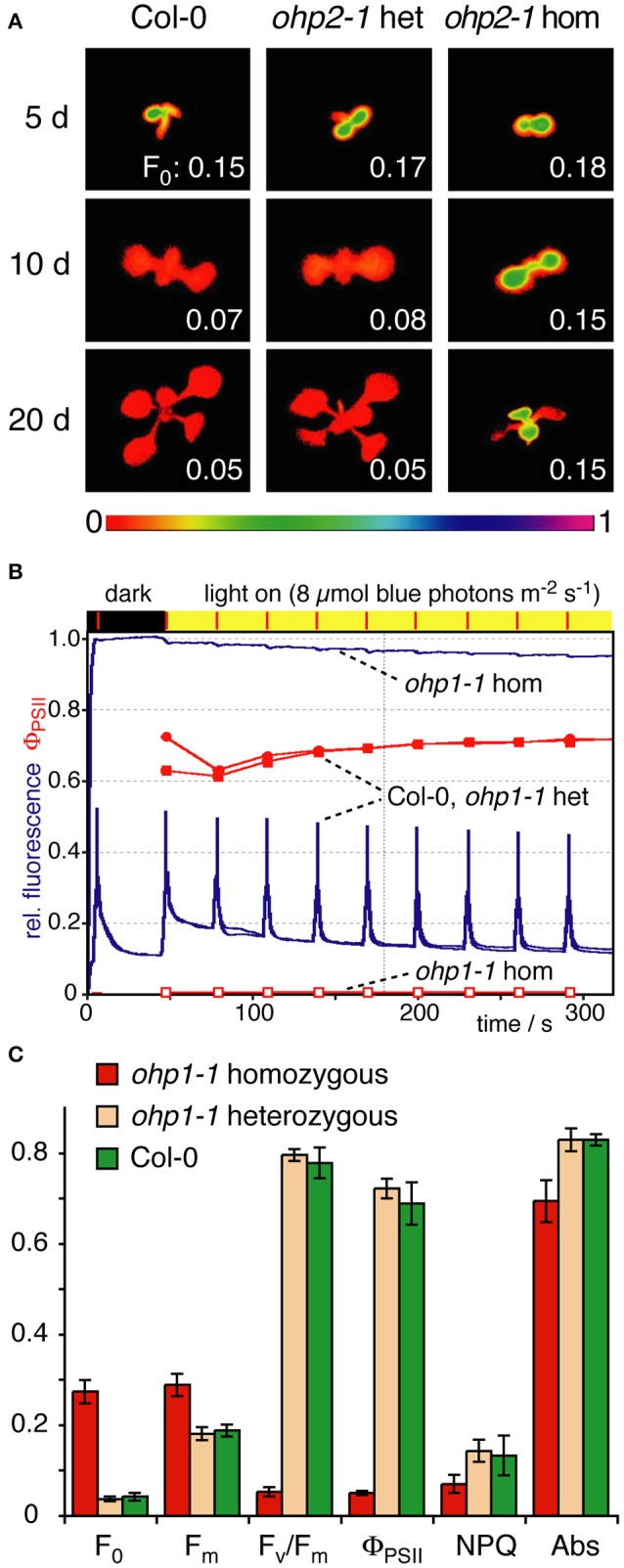
**Photosynthetic performance of *ohp1* and *ohp2* mutants. (A)** F_0_ images of WT plants, heterozygous and homozygous *ohp2* mutants were recorded 5, 10, and 20 d after germination. Plants were grown in a 12 h light/12 h dark cycle at a light intensity of 10 μmol photons m^−2^ s^−1^ at 18°C. The bar below represents the color to numeric value conversion. The F_0_ values indicated in the panels are the average over the area of one cotyledon (5 and 10 d after germination) or a true leaf (at 20 d). **(B)** Fluorescence traces (blue lines) of an induction kinetic using mature true leaves of 3-week-old homozygous and heterozygous *ohp1* mutants as well as WT plants. The plants were exposed to blue actinic illumination of 8 μmol photons m^−2^ s^−1^, which is corresponding to the cultivation conditions with 15 μmol photons m^−2^ s^−1^ white light. The operating efficiency of PSII (Φ_PSII_) is shown in red. **(C)** Fluorescence-derived photosynthetic parameters of mature leaves analyzed as described above. F_0_, Minimal fluorescence of dark-adapted leaves; F_m_, Maximal fluorescence of dark-adapted leaves; F_v_/F_m_, Maximal photochemical efficiency of dark-adapted leaves; NPQ, Non-photochemical quenching; Abs, Relative absorptivity of the leaves. Values are mean±SD (*N* = 5). Homozygous mutants differed significantly from WT and heterozygous plants in all analyzed parameters (Student's *t*-test, *p* < 0.05).

To examine whether reduced pigment content of *ohp1-1* and *ohp2-1* mutants impaired photosynthesis, we monitored Chl fluorescence using pulse amplitude-modulated (PAM) fluorimetry. During de-etiolation all seedlings showed similar levels of ground state fluorescence (F_0_). In WT and heterozygous seedlings F_0_ declined during further development, whereas homozygous *ohp1-1* and *ohp2-1* maintained a high F_0_ that only declined when the leaves bleached and died (Figure 5A and data not shown). Induction kinetics showed typical fluorescence traces in WT plants and heterozygous *ohp* mutants. In contrast, fluorescence emission in homozygous *ohp1-1* and *ohp2-1* mutants was nearly independent of the light intensity (Figure 5B and Supplemental Figure 3). In mature true leaves of homozygous mutants, F_0_ values were six to nine times as high as in heterozygous or WT plants (Figure 5C). Maximal fluorescence values (F_m_) during a saturating light flash were only slightly higher than F_0_ in homozygous *ohp1-1* and *ohp2-1* mutants. F_m_ values were approximately 1.5- or 1.9-fold higher in homozygous *ohp1-1* or *ohp2-1* mutants, respectively, compared to heterozygous mutants and WT plants. Maximum quantum efficiency of PSII in the dark-adapted state (F_v_/F_m_) was close to 0 in homozygous mutants, whereas heterozygous mutants and WT plants showed values close to 0.8 typical for photosynthetically fully competent leaves (Björkman and Demmig, 1987; Johnson et al., 1993). The F_v_/F_m_ values in homozygous *ohp1-1* mutants were highest (up to 0.13) in very young leaves but rapidly declined to 0 with increasing leaf age (Supplemental Figure 3). The quantum efficiency of PSII in the light acclimated state (Φ_PSII_) was typically below 0.1 in leaves of homozygous *ohp1-1* and *ohp2-1* mutants, compared to expected values around 0.7 in heterozygous mutants and WT plants. Upon exposure to high light (150 μmol photons m^−2^ s^−1^), the fluorescence dropped rapidly in photosynthetically competent young leaves of homozygous *ohp1-1* mutants concomitant with a transient increase in NPQ values. In contrast, fluorescence stayed at levels similar to F_0_ in older leaves and NPQ remained low (Supplemental Figure 3).

### PSI and PSII reaction centers are affected by depletion of OHP proteins

**Figure 6:**
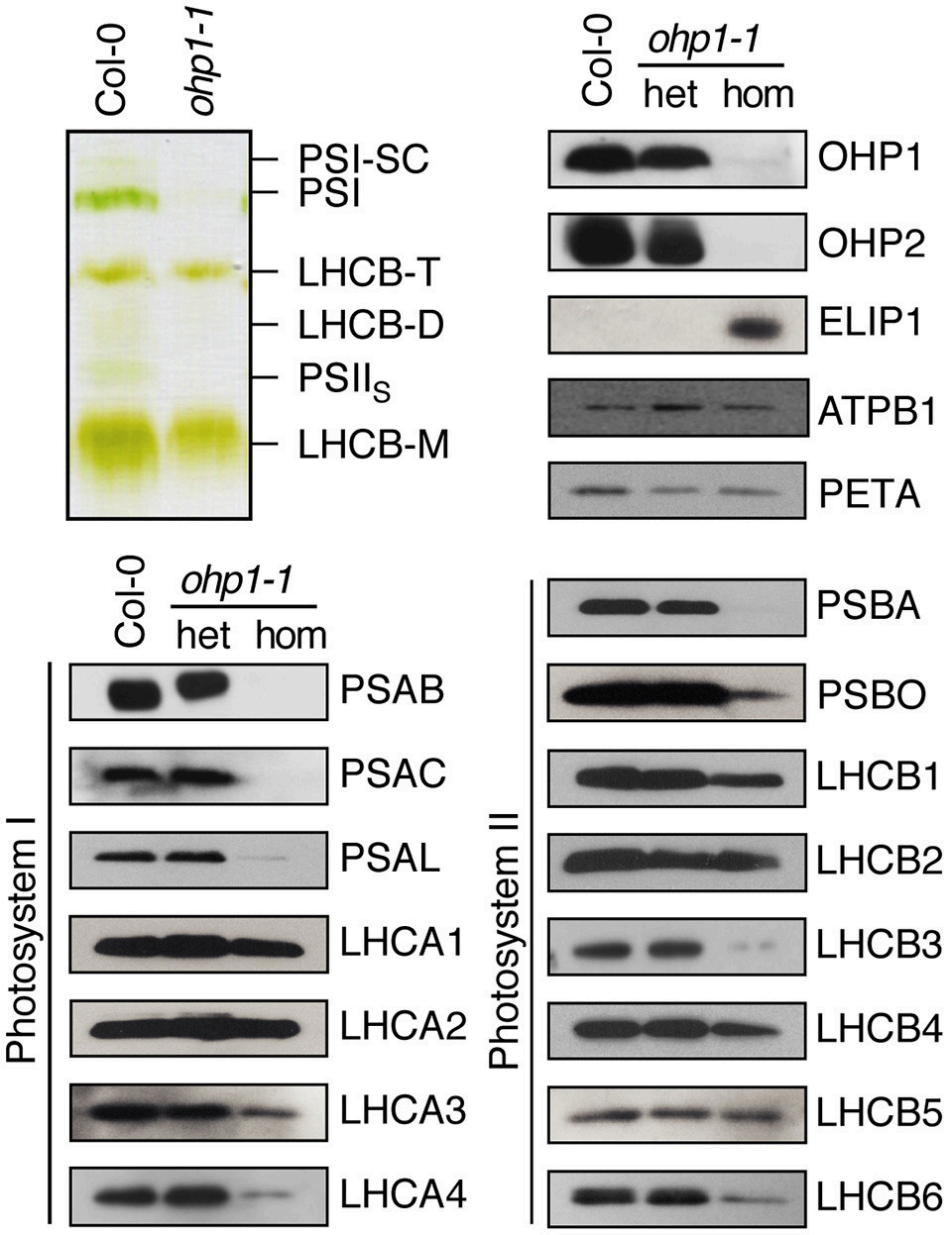
**Deletion of OHP1 affects specific pigment-protein complexes in the thylakoid membrane**. Total membrane protein extracts of entire rosettes of 10-week-old homozygous and heterozygous *ohp1-1* mutants and WT plants cultivated at 15 μmol photons m^−2^ s^−1^ in an 8 h light/16 h dark cycle were analyzed by green native PAGE or Western blotting after denaturing SDS-PAGE. Equal amounts of protein were loaded in each lane.

The similar regulation of *OHP1* and *OHP2* expression and the nearly identical mutant phenotypes indicated that OHP1 and OHP2 are required for PSII function and that both proteins might be functionally linked. Our western blot analysis revealed that only low levels of OHP2 protein were detected in homozygous *ohp1-1* mutants, and likewise, *ohp2-1* mutants lacked OHP1 protein (Figure 3). Comparative analysis of photosynthetic complexes by native gel electrophoresis showed that homozygous *ohp1-1* mutants lack several components of the photosynthetic machinery (Figure 6). To further investigate these defects in both *ohp1-1* and *ohp2-1* mutants, we analyzed the levels of photosynthetic proteins in 10-week-old plants cultivated at 15 μmol photons m^−2^ s^−1^ with a short-day light regime. The abundance of several antenna proteins of PSII and PSI was reduced in *ohp1-1* and *ohp2-1* mutants compared to WT plants (Figure 6 and Supplemental Figure 4). Strikingly, levels of the reaction center (RC) proteins of both PSII (PSBA) and PSI (PSAB) were near or below the detection limit. Furthermore, the 33 kDa-subunit of the oxygen-evolving complex (PSBO) and PSAK, another subunit of PSI, were strongly reduced in *ohp1-1* mutants. These differences became more pronounced when comparing older plants or when plants were grown at an illumination intensity of 50 μmol m^−2^ s^−1^ (data not shown). Interestingly, homozygous *ohp1-1* and *ohp2-1* mutants but not heterozygous mutants or WT plants showed expression of ELIP1, a phenomenon generally associated with light stress (Adamska et al., 1992).

### Loss of OHP1 causes induction of the antioxidant defense

**Figure 7:**
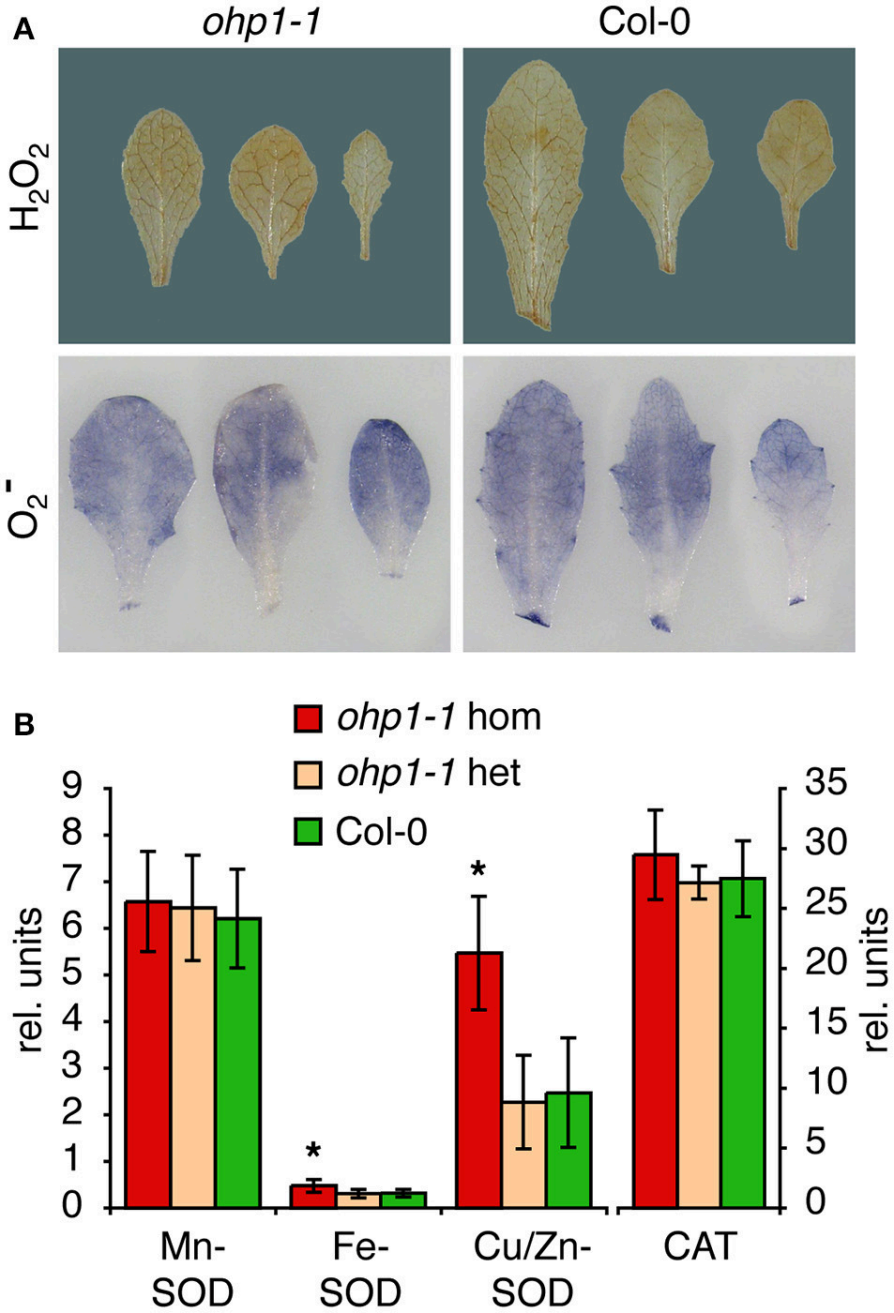
**ROS staining and ROS-scavenging enzyme activities in *ohp1-1* mutants. (A)** Leaves of 4-week-old *ohp1-1* mutants and WT plants cultivated under continuous illumination at 15 μmol photons m^−2^ s^−1^ were stained for H_2_O_2_ content with 3,3-diaminobenzidine or for ${\text{O}}_{2}^{-}$ content with NBT. Leaves were vacuum-infiltrated with staining solution and incubated for 2 h or 20 min, respectively, at 15 μmol photons m^−2^ s^−1^. Three representative leaves of each genotype are displayed. **(B)** Relative activities of the three superoxide dismutase (SOD) isoforms and catalase were quantified in leaf extracts by in-gel staining after native gel electrophoresis. SOD activities were normalized by the total SOD activity of WT leaves for each gel. Values represent the mean ± SD in relative units (*N* = 8 for SODs, *N* = 3 for catalase), asterisks indicate significant difference from the respective activity in WT leaves (*p* ≤ 0.02 in a Student's *t*-test).

The high Chl fluorescence yield and almost complete absence of reaction centers observed in homozygous *ohp1-1* and *ohp2-1* mutants indicated poor usage of excitation energy, which is very likely to cause increased formation of reactive oxygen species (ROS). Staining of leaves with trypan blue revealed that *ohp1-1* mutants did not contain elevated levels of dead cells (data not shown). Staining of leaves for ${\text{O}}_{2}^{-}$ and H_2_O_2_ levels produced very similar pictures for homozygous *ohp1-1* mutants and WT plants (Figure 7). Therefore, we analyzed the activity of superoxide dismutase (SOD) and catalase in leaf extracts. The activities of catalase and Mn-SOD were very similar in WT plants and *ohp1-1* mutants. In contrast, the activities of Fe-SOD and Cu/Zn-SOD were increased in homozygous *ohp1-1* mutants, especially Cu/Zn SOD, for which the activity was increased approximately 2-fold.

### OHP1 and OHP2 are not functionally redundant

**Figure 8:**
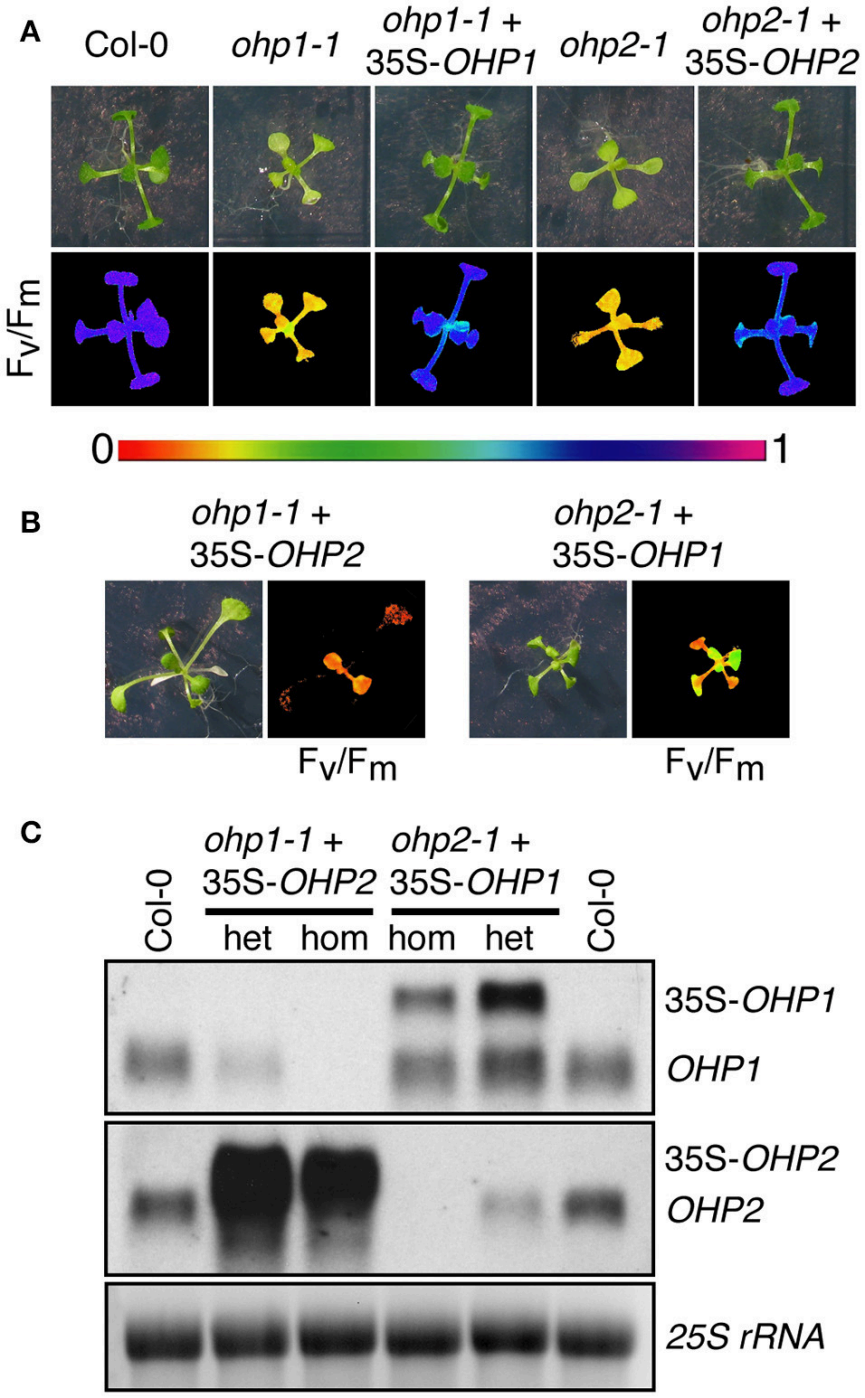
**Complementation of *ohp* mutants. (A,B)** Photos and F_v_/F_m_ images of 2-week-old WT plants, homozygous *ohp1-1* or *ohp2-1* mutants and complemented mutants (T2 generation). Color scale for F_v_/F_m_ is shown between **(A,B)**. In the T1 generation after transformation of heterozygous *ohp* mutants with *OHP* overexpression constructs, plants homozygous or heterozygous for the *ohp1-1* or *ohp2-1* T-DNA insertions were identified by PCR. T2 plants were selected for BASTA resistance conferred by the overexpression constructs, sulfadiazine resistance conferred by the KO-constructs and transferred to plates without herbicide 5 days after germination. **(C)** Transcript analysis of plants from the cross-complementation attempt. Transcripts of the rescue constructs (35S-*OHP1* and 35S-*OHP2*) can be distinguished from endogenous transcripts by their bigger size due to artificial UTRs. Fifteen microgram of total RNA were loaded per lane and EtBR-stained *25S rRNA* is shown as loading control.

The highly similar phenotypes of *ohp1-1* and *ohp2-1* mutants despite the distinct evolutionary origin of both proteins suggested that they might either have a redundant, dose-dependent function or act together in the assembly or maintenance of photosynthetic complexes. To test for functional redundancy and the specificity of the mutant phenotypes, we transformed heterozygous mutants with constructs for overexpression of OHP1 or OHP2. Already in the T1 generation, homozygous *ohp1-1* plants with WT-like appearance were identified in lines carrying a 35S-*OHP1* construct but never with a 35S-*OHP2* construct. In the T2 generation, also the offspring of heterozygous *ohp1-1* mutants carrying the 35S-*OHP1* construct contained homozygous *ohp1-1* mutants that showed normal growth and pigmentation and had a photosynthetic capacity (F_v_/F_m_) similar to WT plants (Figure 8A). Among the offspring of heterozygous *ohp1-1* mutants carrying the 35S-*OHP2* construct, all homozygous *ohp1-1* mutants were pale and showed high Chl fluorescence independent of the presence of the 35S-*OHP2* construct (Figure 8B). Likewise, 35S-*OHP2* but not 35S-*OHP1* complemented the phenotype of homozygous *ohp2-1* mutants. Transcript analysis confirmed that the complementation constructs, which produce longer transcripts due to the insertion of a riboswitch in the 3′-UTR, were expressed even in the lines that failed to show cross-complementation (Figure 8C). These findings demonstrate that the loss of *OHP* expression is the definite cause for the mutant phenotypes and that OHP1 and OHP2 have clearly distinct functions.

## Discussion

### OHP1 and OHP2 proteins are highly conserved

It is striking that all well annotated genomes of phototrophic eukaryotes except dinoflagellates and chlorarachniophytes contain at least one HLIP-encoding (glaucophytes and red algae) or OHP1-encoding (green algae and plants) gene and one gene encoding an OHP2 protein (Engelken et al., 2012). Despite the similar predicted secondary structure with a single transmembrane helix, OHPs are most likely not a monophyletic group of proteins (Figure 1; Engelken, 2010). The phylogenetic tree derived from the alignment comprising the entire sequence of the mature OHP1 protein roughly displays the expected species topology in the green lineage with a gradual evolution via green algal, bryophyte and pteridophyte OHP1 sequences to higher land plants (Rodríguez-Ezpeleta et al., 2005). While OHP1-type proteins were probably directly derived from cyanobacterial HLIP ancestors, OHP2-type proteins are more closely related to the eukaryotic Light Harvesting-Like proteins (LILs) than to OHP1 and HLIPs and have probably evolved by the loss of the second transmembrane helix present in LILs (Figure 1; Engelken et al., 2012).

The broad abundance and strong conservation can be taken as an indication that both OHP1 and OHP2 fulfill essential functions in thylakoids. In contrast to the high diversity of HLIPs and their variable number per organism, OHP1 and OHP2 sequences typically occur as one copy per organism and they are highly conserved, especially in land plants including mosses (Figure 1 and Supplemental Figure 1). *Synechocystis* mutants missing all four HLIPs were not viable while growing under high light conditions and it could be shown that HLIPs in cyanobacteria stabilize PSI trimers under exposure to high irradiance (He et al., 2001; Wang et al., 2008). More recently, cyanobacterial HLIPs were associated with delivery of newly synthesized or recycled pigments during assembly of photosystem II and potentially also Photosystem I (Komenda and Sobotka, 2016). They were found to interact in pairs or as dimers with photosystems and other proteins involved in pigment synthesis and pigment-protein assembly factors (Chidgey et al., 2014; Knoppová et al., 2014; Akulinkina et al., 2015; Staleva et al., 2015). The strong conservation suggests that OHPs in plants fulfill similar functions while some of the functions of HLIPs might be taken over by LILs or ELIPs in plants, thus allowing the reduction to one copy each of OHP1 and OHP2.

### Co-regulation of OHP1 and OHP2 expression suggests a common function

Expression of both OHP1 and OHP2 was found to be regulated at the transcript level in response to different light conditions (Jansson et al., 2000; Andersson et al., 2003; Stawski et al., 2014). Our detailed expression analysis revealed that *OHP1* and *OHP2* transcript levels are co-regulated during the diurnal light cycle, during de-etiolation and in response to excess light (Figure 2). In contrast to *ELIP1* transcripts, *OHP1* and *OHP2* mRNAs were detected under all conditions in photosynthetically active tissues, indicating that the function of OHPs is not limited to light stress protection. Transcript levels of *OHP1* and *OHP2* were increased by approximately 25, 50, and 100% at the beginning of the light phase, in response to high light stress and during de-etiolation of dark-grown seedlings, respectively, indicating that OHP proteins may be especially important when the synthesis or repair rates of photosynthetic protein complexes in the thylakoid membrane are high. A high degree of co-expression across the total range of transcriptome analyses summarized in the AttedII database indicates that co-regulation of *OHP1* and *OHP2* expression may not be limited to light responses (Obayashi et al., 2014). *OHP1* and *OHP2* expression was additionally co-regulated with genes of the IPP metabolism, which is the starting point for isoprenoid biosynthesis (Vranová et al., 2013). Among the isoprenoids, especially the terpenoids like phytols, tocopherols and carotenoids are essential for assembly and functionality of photosynthetic pigment-protein complexes as well as for photoprotection (Domonkos et al., 2013; Mokrosnop, 2014).

### OHP1 and OHP2 are essential for functional photosystems in arabidopsis

Based on expression patterns during light stress, photoprotective functions were proposed for ELIP family members, both in cyanobacteria and higher plants (Heddad et al., 2012). However, detailed information about the molecular and physiological functions of proteins of the ELIP family is still scarce. Most of the functional studies were performed on three-helix ELIPs or two-helix LILs/SEPs in higher plants and on HLIPs in cyanobacteria. Besides light stress protection, interaction studies and mutant analyses indicated functions in pigment synthesis, pigment transfer and recycling as well as pigment protein complex assembly (Heddad et al., 2012; Sinha et al., 2012; Yao et al., 2012; Stawski et al., 2014; Takahashi et al., 2014; Lohscheider et al., 2015; Komenda and Sobotka, 2016).

In this study, we demonstrate that OHP1 and OHP2 play a crucial role in assembly or maintenance of functional photosystems in Arabidopsis thylakoid membranes. The stepwise assembly of photosystem II in higher plants and the repair cycle that enables selective replacement of damaged D1 proteins in photosystem II reaction centers has been analyzed in great detail and many structural and accessory proteins have been identified (Järvi et al., 2015; Lu, 2016). However, none of the studies on these topics have identified a contribution of OHP1 or OHP2. As described in the present study, the depletion of OHP1 and OHP2 led to drastically reduced levels of both photosystem II and photosystem I reaction center proteins (Figure 6) concomitant with impairment or complete loss of photosynthesis in *ohp* mutants (Figure 5 and Supplemental Figure 3). Induction kinetics of Chl fluorescence quenching demonstrated that leaves of *ohp* mutants rapidly lost any detectable photochemical quenching which is in accordance with the loss of reaction centers. Also inducible non-photochemical fluorescence quenching was barely detectable in *ohp* mutant leaves although a fraction of the LHC antenna was expressed at almost the same level as in WT plants. Pigment concentrations in *ohp* mutant leaves were strongly reduced, especially of Chl and β-carotene. Xanthophyll levels were very similar in leaves of WT plants and *ohp1* mutants, indicating that xanthophyll-mediated light harvesting or energy dissipation in the antenna are not the primary targets of OHP action. Despite the low Chl concentration, ground state fluorescence (F_0_) was much higher in *ohp* mutants than in WT plants, indicating that either the antenna had insufficient possibilities to dissipate the excitation energy or that additionally free pigments accumulated in the mutants. Probably the early seedling lethal phenotype has prevented the identification of *ohp* mutants in previous screens for altered Chl fluorescence (Meurer et al., 1996; Shikanai et al., 1999).

The loss of reaction centers and photosystems also resulted in drastic changes of thylakoid ultrastructure in *ohp1-1* mutants (Figure 4). The presence of LHCII antenna probably stabilized the remaining grana stacks, while the absence of stroma lamella and the swelling of the marginal thylakoids clearly indicate that OHP proteins are essential to build or maintain the correct architecture of the thylakoids. Interestingly, the changes observed in an *ohp1* mutant in the L*er* background were different from what we observed in the Col-0-derived mutant (Myouga et al., 2013). It remains to be determined if these difference reflect an ecotype-specific effect or are due to differences in cultivation conditions or the age of the analyzed leaves.

By complementation of the *ohp1-1* and *ohp2-1* mutants we demonstrated that the mutant phenotypes are specific and exclusively caused by the loss of expression of individual OHPs (Figure 8). Cross-complementation was not observed, indicating that OHP1 and OHP2 have separate functions, although they may act together in the same process. The downregulation of OHP1 protein levels in *ohp2* mutants and *vice versa* complicates the assignment of a specific function to either of the OHP proteins in Arabidopsis. To get more detailed information about the molecular function of OHPs in higher plants, the identification of interaction partners and potential pigment binding capacities need to be determined in the future. We aim to use riboswitch-mediated de-stabilization of *OHP* transcripts in our complemented lines to investigate the consequences of OHP depletion in fully developed chloroplasts.

For cyanobacterial HLIPs, homodimer or heterodimer formation has been postulated as a prerequisite of pigment binding (Knoppová et al., 2014; Staleva et al., 2015; Komenda and Sobotka, 2016). Binding of HLIPs to photosystem II reaction centers has been clearly demonstrated, while also an association with photosystem I complexes is still debated (Akulinkina et al., 2015; Komenda and Sobotka, 2016). Mutant analyses and characterization of isolated protein complexes indicate a function of HLIPs in pigment delivery and energy dissipation during early steps of reaction center protein assembly (Komenda and Sobotka, 2016).

In a similar manner a complex comprising both OHP1 and OHP2 may be formed in higher plants, depending on either protein for functionality. Such a complex might not interact strongly with the structural photosystem components but might be essential for pigment delivery to both reaction centers during biogenesis or repair, which would explain the severe mutant phenotype. Loss of one OHP might not only abolish the function of such a complex, but additionally cause de-stabilization of the other OHP protein, which would explain the nearly identical mutant phenotypes. Similar stabilizing interactions have been observed previously for other thylakoid proteins, e.g., Geranylgeranyl Reductase was found to be unstable in the absence of LIL3 proteins in Arabidopsis and point mutations in *Synechocystis* Cytochrome *b*_559_ de-stabilized the entire PS II complex (Hung et al., 2007; Tanaka et al., 2010).

### Oxidative stress in *ohp* mutants

High Chl fluorescence is typically caused by an increased lifetime of excited Chl molecules, which at the same time increases the risk that excitation energy or excited electrons are transferred to molecular oxygen, leading to the formation of singlet oxygen or superoxide radicals, respectively (Pospíšil, 2012). Superoxide is rapidly converted to the more stable hydrogen peroxide either by chemical dismutation or by the action of superoxide dismutases (Alscher et al., 2002). We did not detect elevated levels of superoxide or hydrogen peroxide in *ohp1-1* mutants, indicating that oxidative stress is not the major reason for the seedling lethality of the mutants. Nevertheless, upregulated activity of chloroplastic FeSOD and Cu/ZnSOD activities indicated that ROS levels or ROS signaling were disturbed in *ohp1-1* mutants (Alscher et al., 2002; Pilon et al., 2011). The absence of strong oxidative stress in *ohp* mutants indicates that OHP proteins do not primarily function in protection against excess light stress.

Based on the improved growth of *ohp* mutants by external supply of sugar and vitamins together with the selective absence of reaction centers, we propose that OHP proteins fulfill a crucial function in either assembly or maintenance of photosynthetic reaction center complexes, similar to their evolutionary ancestors, the cyanobacterial HLIPs.

## Author contributions

IA, JB, MR, SA, JL, and DF conceived the study. MR, JB, SA, and DF screened and identified *ohp1* and *ohp2* mutant lines. MR and DF analyzed transcript expression patterns. UA generated OHP-specific antisera. JB and SA carried out immunoblot analysis of thylakoid membrane proteins. JB analyzed pigment-protein complexes by green native gel electrophoresis. JL performed phylogenetic and *in silico* co-expression analysis. MR, JB, and JL performed analysis of pigment content and composition. DF, MR, SA, and JB measured Chl fluorescence parameters in WT and *ohp* mutant plants. DF generated and analyzed complemented mutants. KM provided and analyzed EM micrographs. JB, JL, and DF wrote the manuscript with critical input by all other authors.

## Funding

This work was supported by the German Research Foundation (DFG, grant Nrs. AD92/7-3 to IA and LO2018/2-1 to JL) and the University of Konstanz. The publication of this article was supported by a DFG grant for open access publishing to the library of the University of Konstanz.

### Conflict of interest statement

The authors declare that the research was conducted in the absence of any commercial or financial relationships that could be construed as a potential conflict of interest.
