# Supplementary material for: Small One-Helix Proteins Are Essential for Photosynthesis in Arabidopsis
Source: Front Plant Sci. 2017 Jan 23;8:7. doi: 10.3389/fpls.2017.00007 (PMC5253381; doi:10.3389/fpls.2017.00007)
Supplement: Supplementary file 1 [file Table1.pdf]

Supplemental Table 1: Primers used in this study

OHP1-f	ATGAGCTCGTCGCCGTTATCT	CDS cloning, Nothern probe synthesis, genotyping
OHP1-r	TTATAGAGGAAGATCGAGTCCTT	
OHP2-f	ATGTCAGTAGCTTCACCGATTC	
OHP2-r	TTTGTCTCTGAAACTCCACTGTA	
ELIP1-f	ATGGCAACAGCATCGTTCAACA	
ELIP1-r	TAATCCTCTCTGGTGCTGGAC	
ACT2-f	ACCTTGCTGGACGTGACCTTACTGAT	
ACT2-r	GTTGTCTCGTGGATTCCAGCAGCTT	
OHP1-LP	CGGAGAAAGAGAGCAGTGTAG	Mutant genotyping
OHP1-LP2	GAAATATGAATGGATCGGAGAAAG	
OHP1-RP	ACTCTCTCAGAGCCGTTAGGT	
OHP2-LP	TCAGATGCTCTCAGACAGAAG	
OHP2-RP	TCAGATGCTCTCAGACAGAAG	
GABI-LB	CCCATTTGGACGTGAATGTAGACAC	
35S-3'-f	AATCCCACTATCCTTCGCAAGACC	
OHP1-cf	CCGGTCGCCACCATGAGCTCGTCGCCGTTATC	Complementation constructs
OHP1-cr	TTGTTTATTAGAATTATAGAGGAAGATCGAGTC	
OHP2-cf	CCGGTCGCCACCATGTCAGTAGCTTCACCGATTC	
OHP2-cr	TTGTTTATTAGAATTATTCCAAGTCTAGAATGC	
