# Supplementary material for: Small One-Helix Proteins Are Essential for Photosynthesis in Arabidopsis
Source: Front Plant Sci. 2017 Jan 23;8:7. doi: 10.3389/fpls.2017.00007 (PMC5253381; doi:10.3389/fpls.2017.00007)
Supplement: Supplementary file 2 [file Image1.PDF]

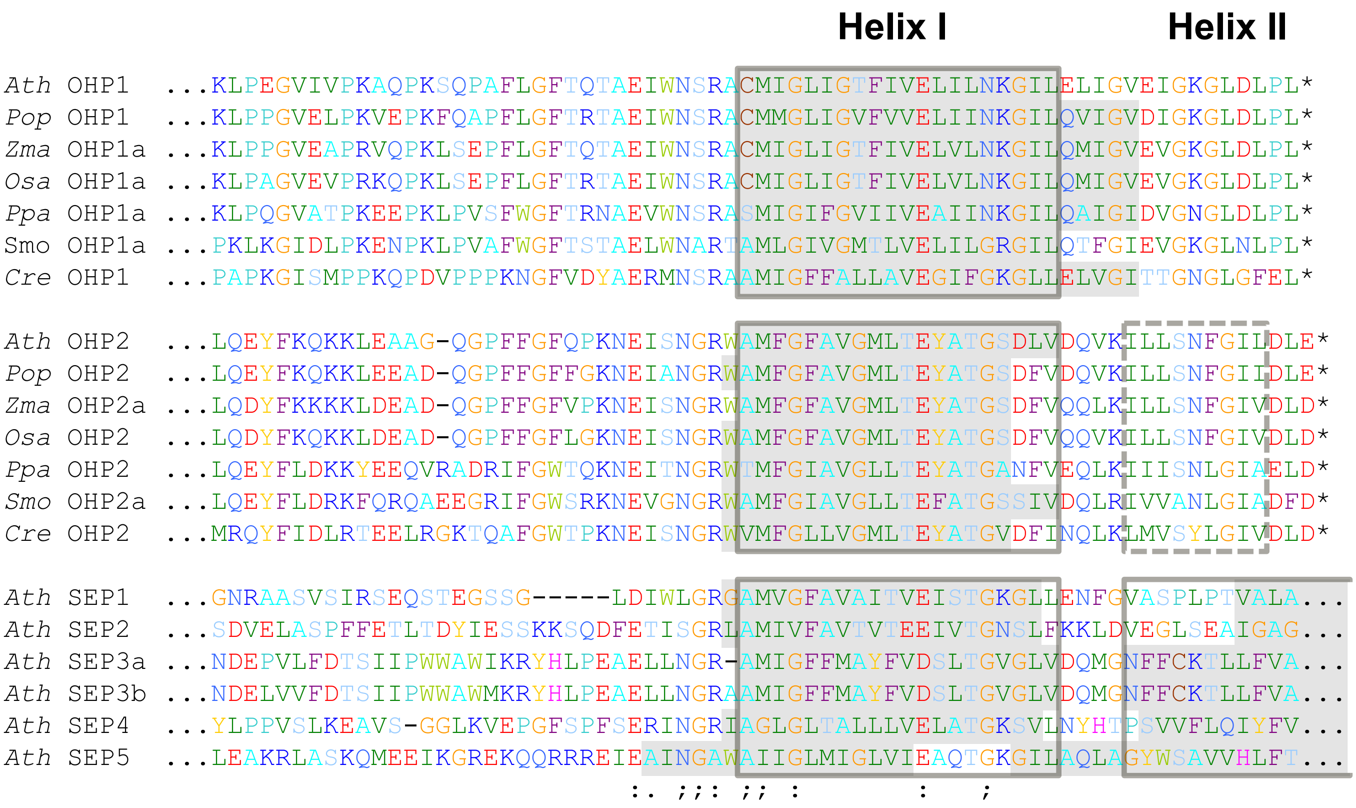

Supplemental Figure 1: Alignment of OHP1, OHP2 and SEP/LIL sequences

OHP1 from Arabidopsis was aligned with the corresponding regions of OHP sequences from selected species of plants, mosses and algae and SEP/LIL proteins from Arabidopsis. Membrane-spanning helices were predicted using HMMTOP (http://www.enzim.hu/hmmtop) and are highlighted in grey and consensus helix regions are boxed. The small hydrophobic region in OHP2 is boxed with a dashed line. Highly conserved residues are marked with :, ; or . in decreasing degree of conservation, * denotes the C-terminus of the protein. *Ath*, *Arabidopsis thaliana*; *Pop*, *Populus trichocarpa*; *Zma*, *Zea mays*; *Osa*, *Oryza sativa*; *Ppa*, *Physcomitrella patens*; *Smo*, *Selaginella moellendorffii*; *Cre*, *Chlamydomonas reinhardtii*.
