# Supplementary material for: Small One-Helix Proteins Are Essential for Photosynthesis in Arabidopsis
Source: Front Plant Sci. 2017 Jan 23;8:7. doi: 10.3389/fpls.2017.00007 (PMC5253381; doi:10.3389/fpls.2017.00007)
Supplement: Supplementary file 3 [file Image2.PDF]

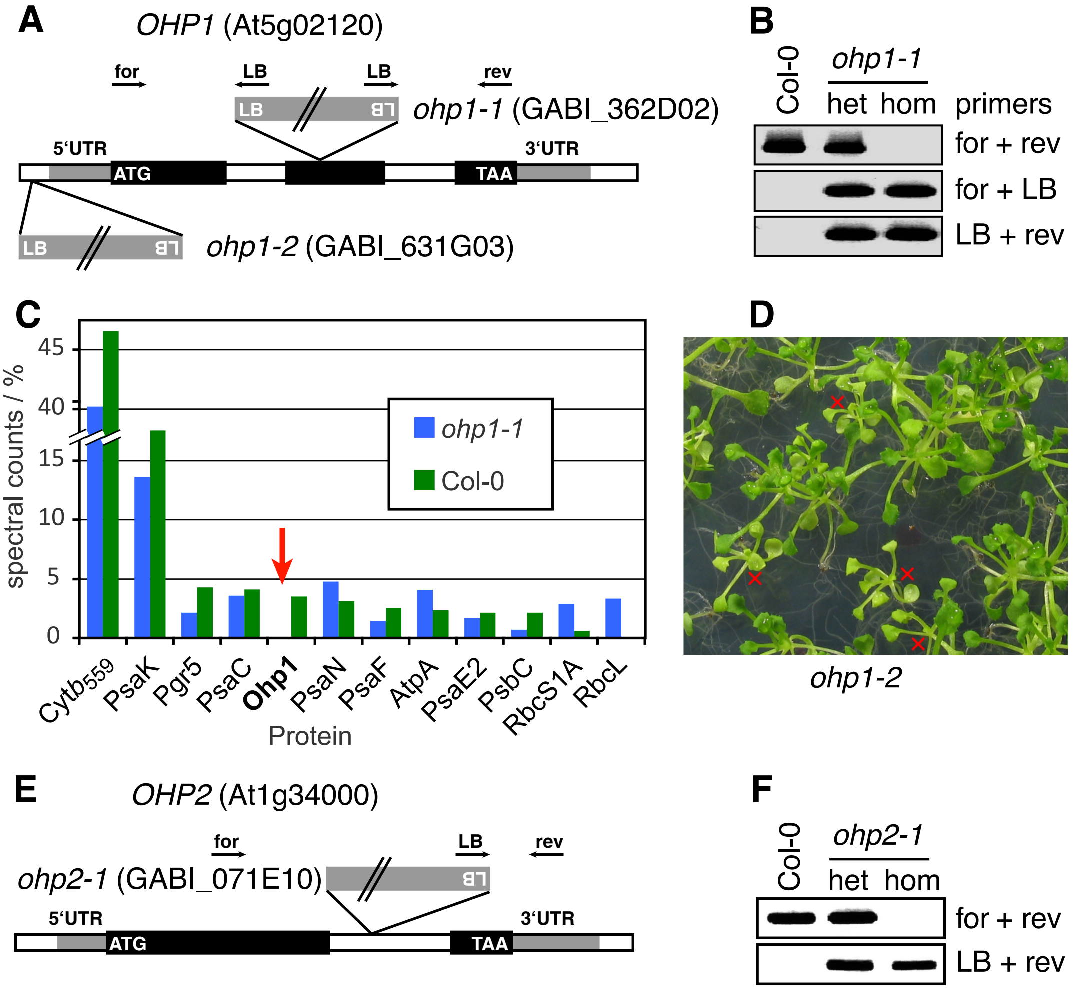

G

line	observed numbers Wt	observed numbers *hcf*	expected numbers Wt	expected numbers *hcf*
ohp1-1	505	99	453	151
ohp2-1	562	123	513.75	171.25

Supplemental Figure 2: Genetic characterization of *ohp1* and *ohp2* mutants

A: Schematic representation of the *OHP1* locus with the positions of the T-DNA insertions in *ohp1-1* and *ohp1-2* mutants. Black boxes represent exons. Arrows indicate the binding sites and orientation of primers used for genotyping. **B** and **F**: Exemplary PCR results of PCR genotyping. **C**: Mass spectrometric detection of tryptic peptides from Col-0 WT plants and homozygous *ohp1-1* mutants. Solubilized membrane proteins were separated on a 15% PAA gel and the region corresponding to 9-11 kDa was analyzed. Numbers of specific peptides were normalized to the total number of peptides identified with high confidentiality. **D**: Phenotype of homozygous *ohp1-2* mutants in comparison to heterozygous and WT plants. **E**: Schematic representation of the *OHP2* locus with the positions of the T-DNA insertion in *ohp2-1* mutants. **G**: Segregation analysis of the high chlorophyll fluorescence (*hcf*) phenotype among the progeny of heterozygous *ohp1-1* and *ohp2-1* mutants. For each mutant line, seed batches of four different plants were analyzed and the total number of plants with the *hcf* phenotype was significantly lower than expected ($p<0.05$ by χ^2-test).
