# Supplementary material for: Small One-Helix Proteins Are Essential for Photosynthesis in Arabidopsis
Source: Front Plant Sci. 2017 Jan 23;8:7. doi: 10.3389/fpls.2017.00007 (PMC5253381; doi:10.3389/fpls.2017.00007)
Supplement: Supplementary file 4 [file Image3.PDF]

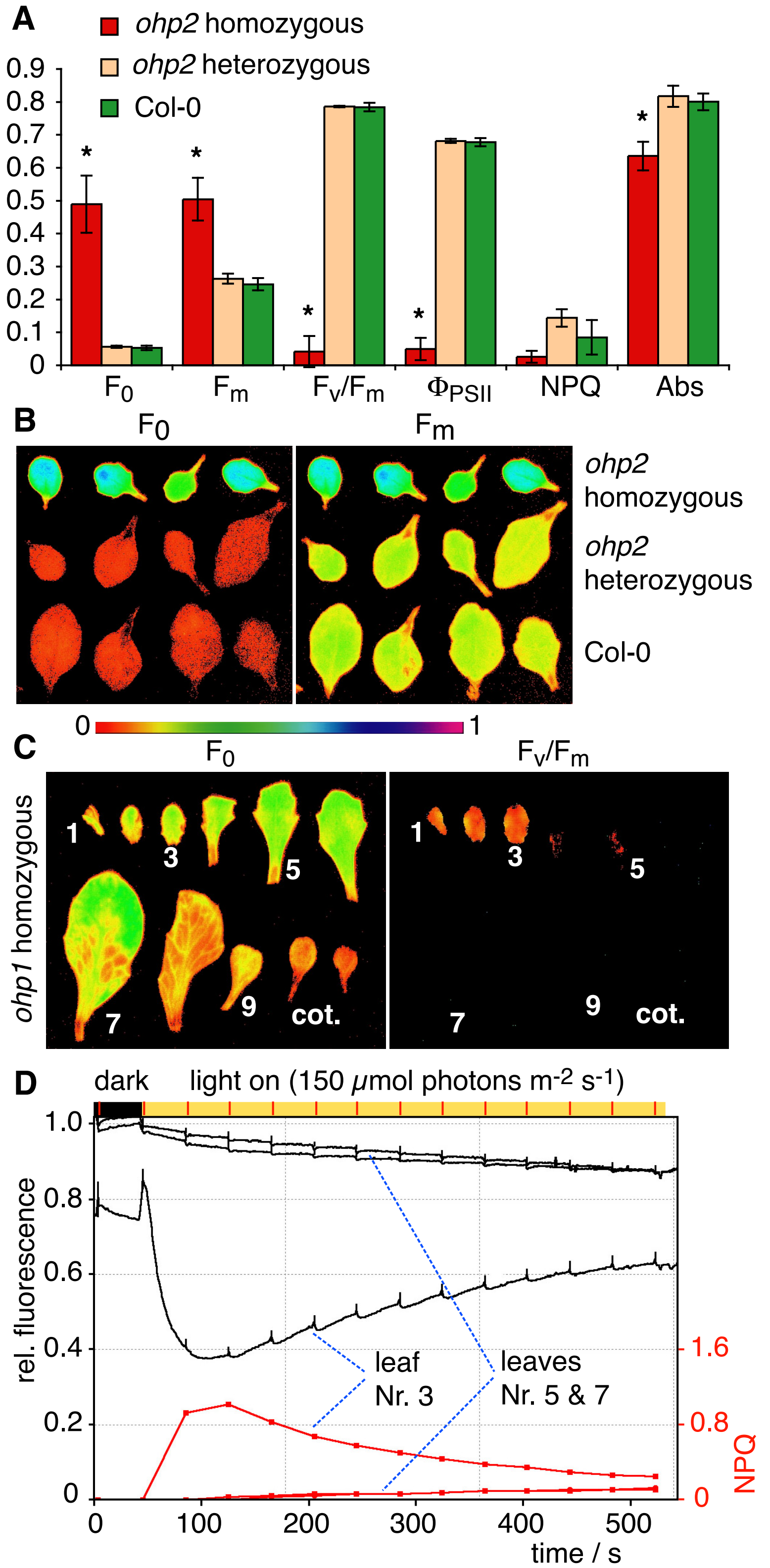

Supplemental Figure 3: Photosynthetic performance of *ohp1* and *ohp2* mutants

A and **B**: Mature leaves of three-week-old homozygous and heterozygous *ohp2-1* mutants and WT plants were analyzed for photosynthetic capacity and photosynthetic performance by monitoring chlorophyll fluorescence and the induction of photochemical and non-photochemical quenching during illumination with blue light (8 µmol photons m^{-2} s^{-1}, which is corresponding to the cultivation conditions with 15 µmol photons m^{-2} s^{-1} white light). **A**: Comparison of various fluorescence parameters: F_0: Minimal fluorescence of dark-adapted leaves, F_m: Maximal fluorescence of dark-adapted leaves, F_v/F_m: Maximal photochemical efficiency of dark-adapted leaves, Φ_{PSII}: Photochemical quantum efficiency of light-acclimated leaves, NPQ: Non-photochemical quenching, Abs: Relative absorptivity of the leaves. Values are mean±SD (N=5). Homozygous mutants differed significantly from WT and heterozygous plants in all analyzed parameters except NPQ. **B**: False color images for F_0 and F_m (normalized to the highest pixel value for F_m). The color scale is indicated below the images. **C** and **D**: Fluorescence analysis of all leaves of a single three-week-old homozygous *ohp1-1* mutant. **C**: False color images of F_0 and F_v/F_m, color scale as in B. Numbers indicate the leaf number starting from the youngest leaf. Cot: Cotyledons. **D**: Transient induction of non-photochemical quenching (red lines) of chlorophyll fluorescence (black lines) in selected leaves of the same plant as in C. The black bar above the diagram indicates dark adaptation. The yellow bar indicates illumination with 150 µmol photons m^{-2} s^{-1} of blue light, which is light stress for leaves of a plant grown at 15 µmol photons m^{-2} s^{-1} white light. Red lines above the diagram indicate saturating light flashes for the determination of F_m or F_m'.
