# Supplementary material for: Small One-Helix Proteins Are Essential for Photosynthesis in Arabidopsis
Source: Front Plant Sci. 2017 Jan 23;8:7. doi: 10.3389/fpls.2017.00007 (PMC5253381; doi:10.3389/fpls.2017.00007)
Supplement: Supplementary file 5 [file Image4.PDF]

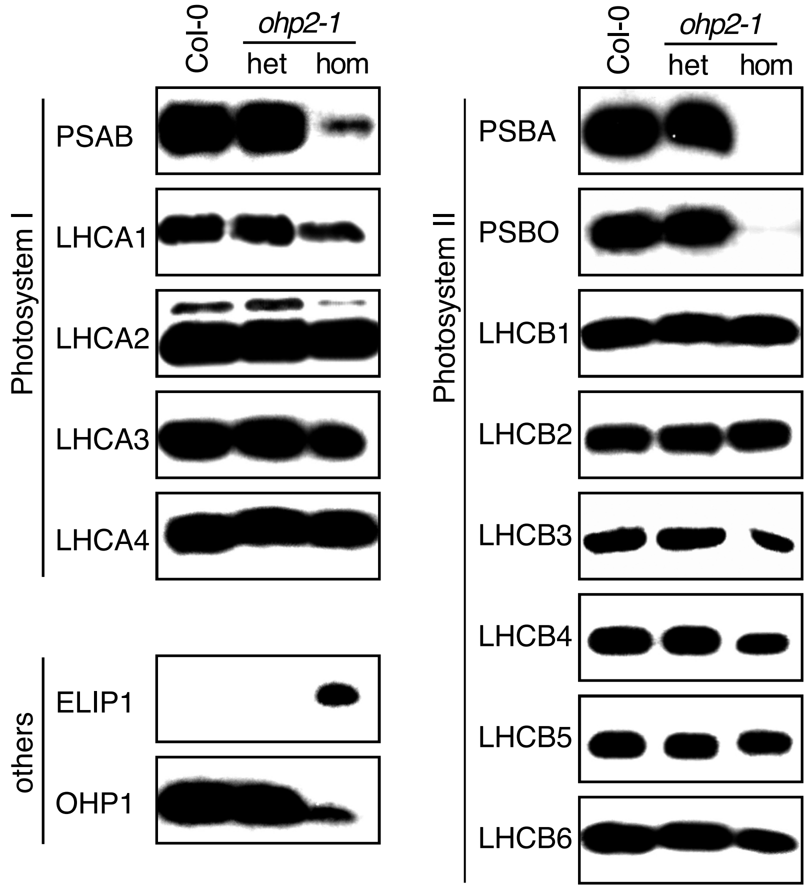

Supplemental Figure 4: Levels of photosynthetic proteins in *ohp2-1* mutants
Immunoblot analysis of protein extracts from leaves of one-month-old WT (Col-0), heterozygous (het) and homozygous (hom) *ohp2-1* mutant plants cultivated in a 8 h light/16 h dark cycle at 25°C and a light intensity of 10 µmol photons m^{-2} s^{-1}. Equal amounts of total protein (15-20 µg) were loaded in each lane.
